# Zinc Uptake by HIV-1 Viral Particles: An Isotopic Study

**DOI:** 10.3390/ijms242015274

**Published:** 2023-10-17

**Authors:** Olivia Guillin, Emmanuelle Albalat, Caroline Vindry, Elisabeth Errazuriz-Cerda, Théophile Ohlmann, Vincent Balter, Laurent Chavatte

**Affiliations:** 1 Centre International de Recherche en Infectiologie (CIRI), 69007 Lyon, France; guillinolivia@hotmail.fr (O.G.); caroline.vindry@gmail.com (C.V.); theophile.ohlmann@ens-lyon.fr (T.O.); 2 Institut National de la Santé et de la Recherche Médicale (INSERM), Unité U1111, 69007 Lyon, France; 3 Ecole Normale Supérieure de Lyon, 69007 Lyon, France; emmanuelle.albalat@ens-lyon.fr; 4 Division Recherche, Université Claude Bernard Lyon 1 (UCBL1), 69008 Lyon, France; 5 Centre National de la Recherche Scientifique (CNRS), Unité Mixte de Recherche 5308 (UMR5308), 69007 Lyon, France; 6 Centre National de la Recherche Scientifique (CNRS), Unité Mixte de Recherche 5276 (UMR5276), 69007 Lyon, France; 7 Center of Quantitative Imagery Lyon Est (CIQLE), Université Claude Bernard Lyon 1, 69008 Lyon, France; elisabeth.errazuriz-cerda@univ-lyon1.fr

**Keywords:** zinc, MC-ICP-MS, isotope fractionation, isotope labeling, viral particles, HIV-1

## Abstract

Zinc, an essential trace element that serves as a cofactor for numerous cellular and viral proteins, plays a central role in the dynamics of HIV-1 infection. Among the viral proteins, the nucleocapsid NCp7, which contains two zinc finger motifs, is abundantly present viral particles and plays a crucial role in coating HIV-1 genomic RNA, thus concentrating zinc within virions. In this study, we investigated whether HIV-1 virus production impacts cellular zinc homeostasis and whether isotopic fractionation occurs between the growth medium, the producing cells, and the viral particles. We found that HIV-1 captures a significant proportion of cellular zinc in the neo-produced particles. Furthermore, as cells grow, they accumulate lighter zinc isotopes from the medium, resulting in a concentration of heavier isotopes in the media, and the viruses exhibit a similar isotopic fractionation to the producing cells. Moreover, we generated HIV-1 particles in HEK293T cells enriched with each of the five zinc isotopes to assess the potential effects on the structure and infectivity of the viruses. As no strong difference was observed between the HIV-1 particles produced in the various conditions, we have demonstrated that enriched isotopes can be accurately used in future studies to trace the fate of zinc in cells infected by HIV-1 particles. Comprehending the mechanisms underlying zinc absorption by HIV-1 viral particles offers the potential to provide insights for developing future treatments aimed at addressing this specific facet of the virus’s life cycle.

## 1. Introduction

Zinc (Zn) is an essential trace element for most living organisms and forms an integral component of numerous enzyme systems. In humans, it ranks as the second most abundant transition metal after iron and is involved in a plethora of biological processes, including but not limited to structural, catalytic, and signaling functions [1]. Zinc is predominantly intracellular (95%), with half of it being found in the cytoplasm, 40% in the nucleus, and 10% associated with the membranes [2,3]. The control of cellular zinc homeostasis is regulated by two prominent families of transporters: the Zrt- and Irt-like proteins (ZIP) and zinc transporters (ZnT). On one hand, ZIP transporters facilitate the influx of zinc ions from the extracellular milieu into the cell, while ZnT transporters mediate the efflux of zinc ions from the cytosol to the extracellular space. On the other hand, these transporters also play a crucial role in maintaining zinc ion homeostasis by facilitating the transport of zinc ions between the cytosol and specific organelles such as the Golgi apparatus, endoplasmic reticulum (ER), and lysosomes [3]. The concentration of cytosolic labile zinc ions, also referred to as free zinc, is maintained at a minimal level, as it may prove harmful for several physiological reactions. As such, metallothioneins regulate this level to the picomolar range [4]. These small cysteine-rich proteins, present in four major isoforms, are key proteins involved in zinc and copper homeostasis in mammals. It is estimated that at least 10% of the proteome is capable of binding zinc [5]. In proteins, zinc can bind to nitrogen, oxygen, and sulfur atoms, enabling its coordination with several amino acids. Zinc-binding proteins play an essential role in cell growth, division, differentiation, cellular signalization, apoptosis, redox homeostasis, membrane structure, transcription, and DNA replication. Zinc finger proteins (ZFs) are a major class of zinc-binding proteins that have non-covalently bound zinc to cysteine and histidine amino acid residues, ensuring proper folding of the protein and facilitating its activity [6,7,8]. Generally, zinc finger domains enable interactions with a variety of biomolecules, such as DNA, RNA, proteins, or lipids, depending on the zinc finger motif. Zinc finger proteins are abundant in eukaryotes, but they are also present in bacteria, archaea, and viruses.

**Figure 1:**
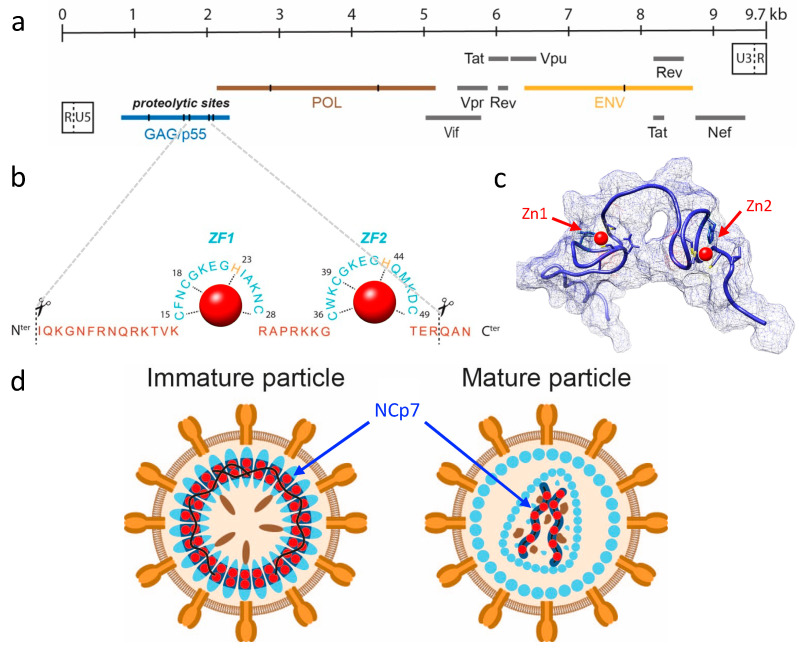
HIV-1 RNA genome and particle structure. (**a**) Genomic organization of HIV-1 RNA based on the three reading frames. The 9.7 kb mRNA is flanked by long terminal repeat (LTR) sequences at the 5′ and 3′ ends. The HIV-1 genome encodes 15 viral proteins and 2 peptides expressed by 9 ORFs. Among them, three encode the polyproteins GAG (in blue), GAG-POL (in brown), and ENV (in orange). The GAG precursor encompasses viral structural proteins, including matrix (MA or p17), capsid (CA or p24), nucleocapsid (NCp7), p6, and two spacer peptides. In addition to the major proteins, the HIV-1 genome codes for six accessory proteins: Vif, Rev, Tat, Nef, Vpu, and Vpr [13]. (**b**) Primary sequence of the NCP7 protein with the two zinc finger domains indicated (ZF1 and ZF2). The coordination of the two zinc atoms (in red) with the CCHC residues of the two domains is also shown. (**c**) A 3D structure of the NCP7 protein in blue (PDB: 1F6U) represented in backbone and surface form, with the positions of the two zinc atoms indicated in red. (**d**) Diagram illustrating the organization of mature and immature HIV-1 particles, with the location of the NCP7 protein in blue and the zinc atoms in red.

The human immunodeficiency virus (HIV) is an enveloped, linear, positive-sense single-stranded RNA virus belonging to the Retroviridae (Group VI) family under the genus *Lentivirus*. HIV is the etiological agent of acquired immunodeficiency syndrome (AIDS), and it targets and infects immune cells, ultimately leading to immunosuppression. With roughly 1.5 million deaths attributable to AIDS each year, HIV infection is a critical global health issue. HIV has two types, HIV-1 and HIV-2, which vary in their epidemiology and pathogenesis. While HIV-2 is largely limited to West Africa, HIV-1 has spread worldwide, owing to its high infectivity and virulence. The HIV-1 genome, which comprises roughly 9.7 kilobases of genomic RNA (gRNA), is reverse transcribed into DNA upon entering the host cell, followed by integration into the host genome to produce new infectious particles. The viral life cycle is intricate, subject to extensive transcriptional and translational regulation, and involves interactions with numerous host cell components [9]. As illustrated in Figure 1a, the HIV-1 genome encodes 15 viral proteins and 2 peptides expressed from 9 open reading frames (ORFs), each with specific roles in the infection process. Among these proteins, nucleocapsid (NCp7), a small structural protein of 55 amino acids (7 kDa), is generated via sequential processing of the p55 Gag precursor polyprotein (Pr55^gag^; see Figure 1b,c). NCp7 is involved in multiple stages of HIV-1 replication, such as reverse transcription of gRNA, integration of DNA into the host genome, and recruitment of two gRNA molecules into viral particles (Figure 1d) [10,11]. NCp7 contains two CCHC zinc-finger motifs, which are required for its nucleic acid binding activities. It is estimated that each HIV-1 viral particle contains approximately 5000 NCp7 molecules, equating to 10,000 zinc atoms [12]. Given the inner diameter of a spherical HIV-1 virion (approximately 100 nm) and its volume of roughly 4.2 × 10^−18^ L, a high intra-particle zinc concentration is inferred to be in the order of 4 millimolar, rendering this element one of the most abundant inorganic components in HIV-1 particles.

Zinc is a mixture of five stable isotopes, which differ solely in their neutron number and exhibit varying relative abundances: ^64^Zn (48.268%), ^66^Zn (27.975%), ^67^Zn (4.102%), ^68^Zn (19.024%), and ^70^Zn (0.631%). As long employed by geologists, the isotopic ratios of a particular chemical element can display distinct behaviors during physical, chemical, or biochemical processes, leading to variations in the isotopic ratios [14,15,16]. Over the past two decades, a rising number of reports have indicated isotopic fractionation in biological processes, particularly for calcium, iron, copper, and zinc [17,18,19,20,21,22]. Zinc isotope fractionation has been identified in various physiological [23,24] and pathological conditions [24,25,26,27], including the brains of Alzheimer’s and Creutzfeldt–Jakob disease-specific mouse models, relative to control mice. Moreover, in humans, the zinc isotopic composition of blood became increasingly enriched with heavy isotopes with aging, as reported in [23]. While the exact reasons for the isotopic fractionation of zinc remain unknown, it is suggested that variations in the binding of zinc to various zinc finger proteins and metallothioneins, which exhibit different expressions based on the tissue or its pathophysiological state, may be a contributing factor. Ab initio calculations have demonstrated that heavy zinc tends to be enriched in complexes with histidine relative to cysteine [28]. In biology, the molecular configuration, redox conditions, and kinetics are known to be the primary driving forces for isotopic variations [15,22]. Since zinc does not participate in redox reactions, the sole factors influencing its isotopic fluctuations encompass molecular binding as well as transportation across membranes, either through active mechanisms (ZIP or ZnT) or passive diffusion. Therefore, zinc is an element that exhibits a relatively low range of isotopic fractionation.

In addition to natural isotopic fractionation, the use of isotopes for labeling purposes has played a critical role in biochemical research, particularly in elucidating various metabolic pathways [21,29]. Isotope exchange and all other applications of isotopes for labeling purposes rely on the assumption that isotopic substitution of an atom in a molecule does not affect its chemical properties and that any effect on its kinetic properties is sufficiently small to be neglected in the analysis. Conversely, the analysis of isotopic effects assumes that measurable differences exist between the kinetic or equilibrium properties of molecules substituted with isotopes. The mass difference between the isotopes of a chemical element can indeed be sufficient to modify the physical and chemical properties of molecules made from different isotopes. In the case of zinc and other transition metals, the analysis of isotopic effects on biological or biochemical activity has never been reported to the best of our knowledge.

The replication and infectivity of HIV-1 virus critically depend on the activity of its zinc finger protein NCp7, which itself relies on the presence of zinc atoms [11]. Even slight perturbations of the zinc finger modules of NCp7 can induce detectable changes in its viral activity since it is involved in multiple stages of viral replication [30]. Therefore, this model would be highly relevant for investigating a potential isotopic effect of zinc on the function of a zinc finger protein by following the viral production and infectivity of the produced particles. Initially, our work aims at determining whether viral particles can recruit enough zinc to measure isotopic fractionation and compare them to cells and a culture medium. In addition, we have designed and developed an experimental protocol to enrich cells with specific zinc isotopes in order to measure the impact of isotopically enriched zinc on the production, morphology, and infectivity of HIV-1.

## 2. Results

### 2.1. Zinc Is Recruited into HIV-1 Virus Particles via the Zinc Finger Domains of NCp7

Our primary objective was to develop an experimental strategy capable of detecting and quantifying the presence of zinc within HIV-1 particles in order to establish a link between this transition metal and the viral protein NCp7. One of the major hurdles that we first encountered was that zinc could not be easily analyzed via ICP-MS due to its significant presence in many laboratory chemicals and labwares. For instance, we found that commonly used products like sucrose, NaCl, and Tris, which are used to prepare the sucrose cushion for viral particle concentration via ultracentrifugation, often contain high zinc levels. Therefore, we selected chemicals with the lowest zinc content for use in virus production, purification, and subsequent analysis via ICP-MS. We also refined our handling of cells and viruses during sample collection; we used plastic and polytetrafluoroethylene (PTFE) supplies that were pre-cleaned as described in the Materials and Methods section.

We anticipated that the majority of the zinc present in HIV-1 particles was bound to the viral structural protein NCp7, as the levels of other zinc-binding proteins in the viral particle, such as integrase, tat, and cellular proteins, were significantly lower. Assuming an average of 5000 NCp7 molecules per virus particle, with each protein possessing two zinc atoms attached to its zinc finger domains, a total of 10,000 zinc atoms per virus particle can be anticipated [12]. Consequently, each particle contains a minimum of 1.9 × 10^−21^ kg of zinc, given that the mass of a single zinc atom is 1.9 × 10^−25^ kg. Nonetheless, the quantification limit (LOQ) achievable with our iCAP-Q ICP-MS extended to levels to the order of 10^−12^ kg, thus requiring a minimum of 10^9^ viral particles for detection and quantification of the zinc in these particles. We produced HIV-1 particles through transfection of HEK293T cells using the pNL4-3 plasmid, which enabled large-scale production and the synchronous release of a high number of HIV-1 particles in the culture media 48 h post transfection [31]. By employing anti-p24 ELISA assays, we estimated viral production to be approximately 10^4^ particles per HEK293T transfected cell in 48 h. This corresponded to a total of 2.6 × 10^10^ HIV-1 particles generated from a starting population of 1.5 × 10^7^ HEK293T cells cultured in a plate 15 cm in diameter.

**Figure 2:**
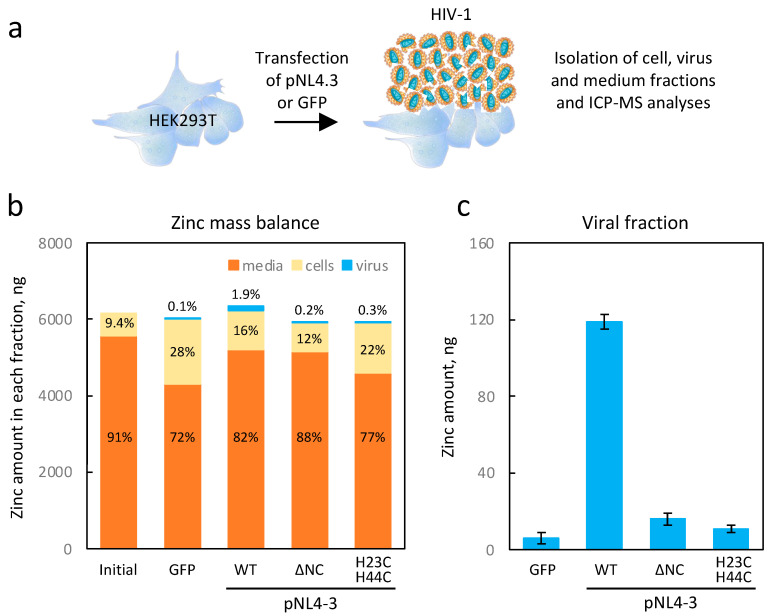
Zinc is recruited in HIV-1 virions via the NCP7 proteins. (**a**) The experimental design involved virus production in HEK293T cells. The cells were transfected with the pNL4.3 plasmid (in a dish 15 cm in diameter), and 48 h post transfection, different fractions were collected and analyzed using ICP-MS. (**b**) The histogram displays the quantities of zinc (in ng) measured in the various fractions (medium, cells, and virus) under different experimental conditions. In addition to the wild-type pNL4.3 plasmid, two mutants (∆NC and H23C/H44C) were employed. A transfection of the GFP plasmid was used as a control. The percentages of the different fractions are indicated on the bars themselves. (**c**) The histogram represents the zinc content in the viral fraction.

As depicted in Figure 2a, we prepared all fractions for ICP-MS analysis as described in Section 4. As for the initial conditions, we had the fresh culture media and untransfected cells. Two days post transfection, we harvested the cells, the concentrated virus, and the remaining growth media. As shown in Figure 2b, under the initial conditions, about 9% of the total zinc amount was present in the cellular fraction, while the remaining 91% was in the culture medium. After 48 h of viral production, the cells had undergone at least two divisions and contained 16% of the total zinc. The viral fractions contained 119 ng of zinc (Figure 2c), representing 1.9% of the total amount and 11.6% of the producing cells (Figure 2b). Considering the number of particles produced (i.e., 2.6 × 10^10^), we estimated the number of zinc atoms to be approximately 4 × 10^4^ per particle, close to the expected amount due to the presence of 5000 NCp7 per viral particle (i.e., 10^4^ zinc atoms). In conclusion, these initial experiments confirm that a large proportion of zinc in the HIV-1 particles was associated with the presence of NCp7, consistent with our initial hypothesis. 

To validate the association between zinc in HIV-1 particles and the presence of zinc fingers in the NCp7 protein, we employed two pNL4-3-derived constructs containing distinct mutations in this gene (Figure 2), which were previously functionally well characterized [30]. The first mutant, named ∆NC, completely deleted the NCp7 coding region, resulting in the production of only a few noninfectious particles. The second one, H23C/H44C, contained mutations in both the zinc finger motifs that eliminated their ability to bind to zinc, leading to the production of viral particles that were noninfectious and had a defect in their conical shape of the viral core morphology [30]. To confirm the phenotypes of these two plasmids, we conducted a Western blot against p24, as depicted in Supplementary Figure S1. The majority of the Gag protein, including NCp7, remains intracellular in the absence of NCp7 in the ∆NC mutant, leading to negligible amounts of viral particles being produced. On the other hand, the H23C/H44C double mutant produces viral particles at approximately half the level of the wild type but with an observable defect in the maturation process, as previously reported [30]. Using the aforementioned mutants, we isolated the viral fraction from the culture medium using a sucrose cushion and determined the quantity of zinc present in these fractions (Figure 2). The results revealed a nearly 90% reduction in the total zinc content of the viral fractions produced by the two mutants, confirming that most of zinc contained in the viral particle was carried by the zinc finger domains of NCp7.

### 2.2. Light Zinc Isotopes Accumulated in Cells and in the Virus Particles

**Figure 3:**
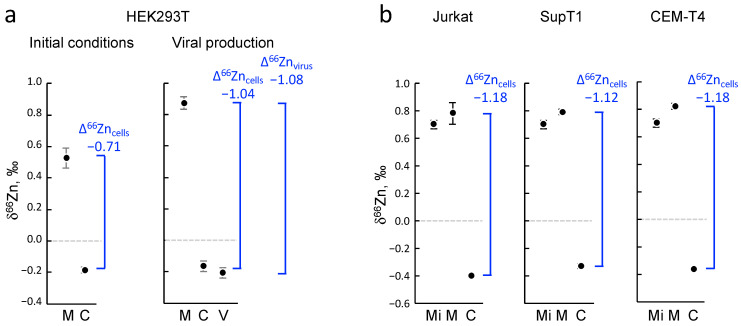
Isotopic fractionation of zinc in different cells and in HIV-1 viral particles. When zinc isotopic fractionation values (δ^66^Zn) exhibit positive results, it signifies an accumulation of heavier isotopes, while negative values indicate a prevalence of lighter isotopes. The ∆^66^Zn values represent differences between two different δ^66^Zn values. (**a**) Zinc isotopic fractionation in HEK293T cells before and after HIV-1 viral particle production. Zinc isotopic fractionation (δ^66^Zn) was measured in the different fractions (n = 3). The ∆^66^Zn values for cells and the virus are indicated in blue. (**b**) Zinc isotopic fractionation in Jurkat, SupT1, and CEM-T4 cell cultures. M = medium after cell growth; Mi = medium at initial conditions; C = cells; V = virus.

After confirming that the presence of zinc in viral particles was indeed associated with the viral protein NCp7, we investigated the isotopic fractionation in relation to the cellular culture and viral replication of HIV-1. More specifically, we analyzed the zinc isotopic composition in the different compartments of the system, namely the culture medium, the HEK293T cells, and the viral fraction, and we compared them to the initial conditions as illustrated in Figure 3. Through isotopic fractionation, we examined whether the cells or viruses produced by these cells exhibited a preference for the light or heavy isotopes of zinc. In order to accomplish this, the three most abundant isotopes of zinc (i.e., ^64^Zn, ^66^Zn, and ^68^Zn) were measured via MC-ICPMS in the samples and compared to a standard reference. Zinc isotope compositions are usually expressed as δ^66^Zn, defined as the measured isotopic ratio in a sample with respect to an international standard solution. Positive values for zinc isotopic fractionation values (δ^66^Zn) indicate an enrichment of heavy isotopes, whereas negative values indicate enrichment of light isotopes. In Figure 3, the δ^66^Zn values of the different compartments are shown, and it can be observed that under the initial conditions, the cells showed a significant enrichment of light isotopes of zinc compared with the culture medium. When using ∆^66^Zn as the differential between the two δ^66^Zn values (medium and cells; Figure 3a), the value was −0.71‰ (Figure 3a). After viral production, we observed that the δ^66^Zn of the viral particles was close to the value measured in the cells, with a slightly lower but significant value (Figure 3a). At the same time, since the cells went through several divisions and preferentially used light isotopes, the medium became enriched with heavy isotopes of zinc. The ∆^66^Zn value between the cells and culture media, which is defined as the difference between two δ^66^Zn values (Figure 3a), was measured to be −1.04‰ when the cells were harvested (Figure 3a). In parallel, the ∆^66^Zn value between the viral fraction and culture media was measured to be −1.08‰ (Figure 3a). In conclusion, our findings demonstrate that cellular growth led to an enrichment of the light isotopes of zinc in cells, while the culture media became enriched with heavier isotopes through mass conservation. Additionally, the viral particles exhibited an isotopic fractionation pattern that closely resembled that of the producing cells.

We then investigated whether this intracellular enrichment of light isotopes was dependent on the type of cell used. Since HIV infects and replicates in CD4+ lymphocyte cells, we examined the isotopic fractionation in cellular models commonly used in HIV-1 studies: Jurkat, SupT1, and CEM-T4 cells. Unlike adherent HEK293T cells, all these lymphocyte cell lines are grown in suspension and in a slightly different culture medium. Ten million cells were cultured for 3 days, and then both the culture medium and the cell fractions were harvested and analyzed through ICP-MS to determine the zinc isotopic distribution as previously described. We observed that, like the HEK293T cell cultures, CD4+ lymphocytes were enriched with light isotopes, while the medium showed a slight enrichment of heavy isotopes. As shown in Figure 3b, the ∆^66^Zn values between the cells and media were similar in the Jurkat, SupT1, and CEM-T4 experiments, and this value appeared to be significantly higher than that observed in the HEK293T cells (Figure 3b). Unfortunately, viral production was much more challenging in the lymphocytes compared with the HEK293T cells due to a weaker production rate, the requirement for a complete replication cycle, and a low proportion of infected cells. The production of HIV-1 virus, being a lentivirus, was not sufficiently efficient in lymphocytes in vitro, even with high multiplicity of infection (MOI), to enable the reliable collection of a sufficient quantity of viruses for accurate zinc measurement. Furthermore, HIV-1, being unstable in the culture medium for an extended period, would require a relatively short harvesting time, resulting in a lower virus yield because lymphocytes do not replicate the virus at the same rate. These technical hurdles hindered our ability to optimize synchronous and efficient viral particle production in sufficient quantities required for measurement via MC-ICP-MS. Despite several attempts, we were unable to accurately measure the isotopic composition of the viruses produced from lymphocytes. As a conclusion, our results showed that the grown cells were enriched with light isotopes of zinc and made the media concentrate heavier isotopes in return. That aside, the viruses seemed to adopt an isotopic fractionation extremely close to that of the producing cells.

### 2.3. Intracellular Zinc in HEK293T Cells Can Be Rapidly Exchanged with Enriched Isotopes

**Figure 4:**
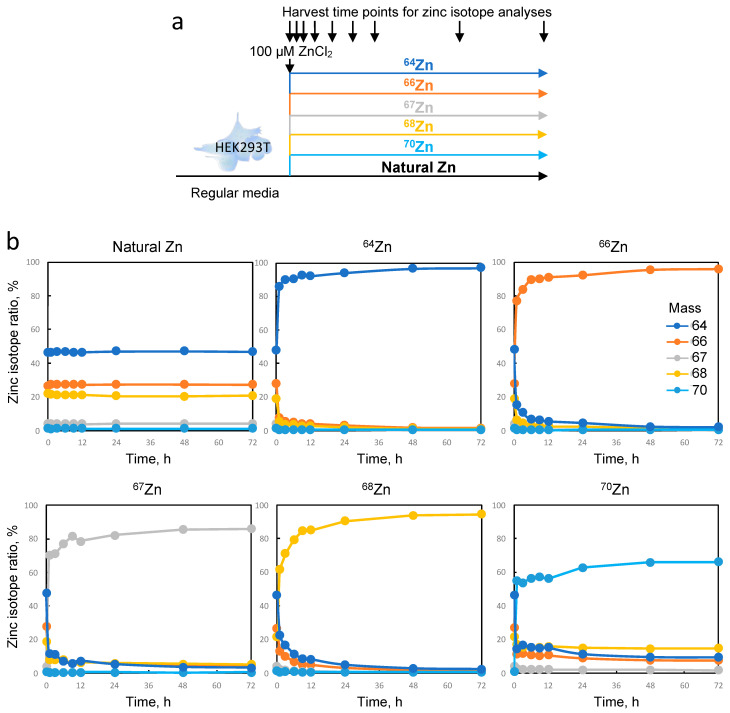
Kinetics of zinc isotope enrichment in HEK293T cells. (**a**) Experimental design. Initially, the cells were seeded and allowed to grow in a standard medium for 1 day. Subsequently, the medium was replaced with one containing 100 µM of the respective ZnCl_2_ isotope or natural zinc. Cell samples were collected at various time points (0, 1, 3, 6, 9, 12, 24, 36, 48, and 72 h). (**b**) Isotopic ratios were determined in all cellular extracts and plotted against time (in hours) for the different medium conditions.

The second aspect of our study aimed to investigate whether the isotopic composition of zinc in NCp7 could significantly alter its activity, as this viral protein is involved in various aspects of virus replication, and even a slight perturbation in its activity can impact viral titer and infectivity [30]. To address this, we decided to produce viral particles enriched with a single zinc isotope using HEK293T cells. Before proceeding, we needed to establish the conditions for isotopic labeling of the cells. Commercially available zinc isotope spikes were used for this purpose in the form of ZnCl_2_. It is worth noting that the availability, purity, and cost of isotopes may vary depending on their natural abundance. In our experiment, the following isotopes were employed, and their respective purities were assessed using single-quadripole ICP-MS: ^64^Zn (99.3%), ^66^Zn (98.9%), ^67^Zn (89.5%), ^68^Zn (98.2%), and ^70^Zn (70.7%). For more information, see Supplementary Table S1. Our initial experiment aimed to determine the time required for the efficient loading of HEK293T cells with the different zinc isotope spikes. In each experimental condition, we supplemented the culture medium (which contained 3 µM of naturally distributed zinc from the FBS) with 100 µM of each isotope spike (Figure 4a). Please note that the first toxic effects of zinc on cell growth were observed above 300 µM. The cells were subsequently collected at regular intervals and analyzed using ICP-MS to determine their isotopic compositions. To the best of our knowledge, the rate of zinc exchange in cultured cells has not been reported to date, which is why we conducted a 3 day kinetics study. As shown in Figure 4b, cellular exchange with each zinc isotope was rapid and nearly complete at 6 h after the addition of the enriched isotope. A plateau was reached in each case, with the following levels of cellular enrichment for each isotope: ^64^Zn (96.9%), ^66^Zn (95.9%), ^67^Zn (86.0%), ^68^Zn (94.5%), and ^70^Zn (66.2%). These levels were slightly lower than the initial purity of each isotope, as we needed to consider the presence of the 3 µM of natural zinc in the starting culture medium, which could not be removed. In conclusion, our results indicate that a rapid and efficient change in the isotopic distribution of zinc can be experimentally induced within a few hours in HEK293T cells using a nontoxic concentration of 100 µM of ZnCl_2_.

### 2.4. HIV-1 Viral Particles Produced in Zinc Isotope-Enriched Cells Were Similar in Quantity, Size, and Infectivity

**Figure 5:**
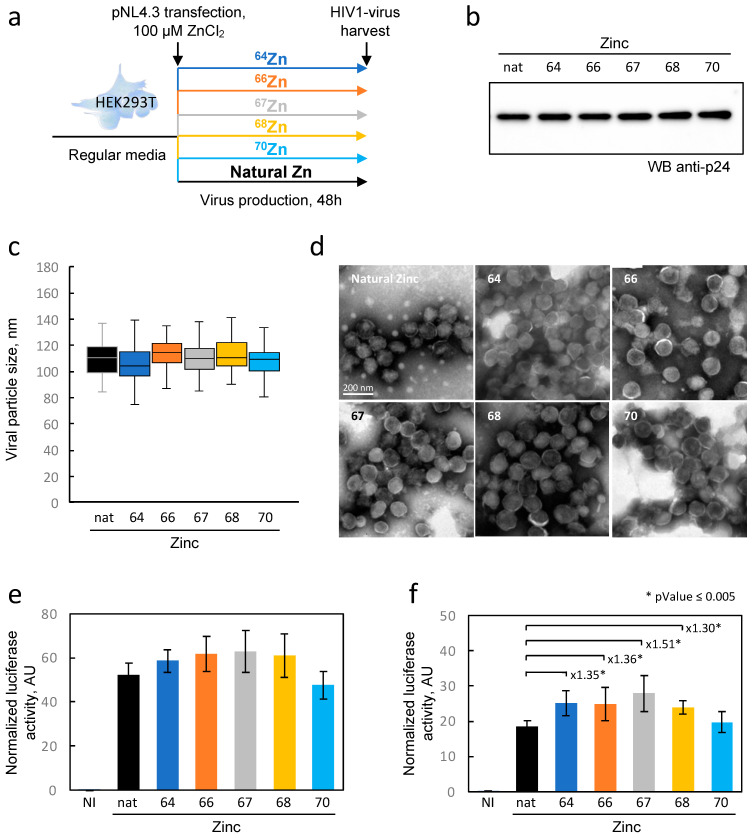
Effect of zinc isotope enrichment of HEK293 cells on the production, morphology and infectivity of HIV-1 particles. (**a**) Experimental design. HEK293T cells were cultured in standard medium and subsequently transfected with the pNL4.3 plasmid. After 4 h, the medium was replaced twice with the one containing the specified isotope every 6 h, as described in Section 4. Two days later, HIV-1 particles were harvested and analyzed for the following parameters: production levels assessed by anti-p24 Western blot (**b**), viral particle sizes analyzed by TEM (**c**,**d**), and particle infectivity tested by infecting TZM-bl cells with two virus dilutions (**e**,**f**). Representative TEM images of the viruses are provided for each isotope in panel D. The particle sizes (n between 80 and 120) are represented in a box-and-whisker plot. For virus infectivity quantification, 2 virus concentrations (stock and ½ dilution) were used to infect TZM-bl cells (e and f, respectively). Luciferase values were normalized to cell density. The means of experimental replicates (n = 6) are depicted with error bars representing the standard deviation values. Nat = natural zinc; NI = not infected control; AU = arbitrary units; asterisk, *p* Value ≤ 0.005.

We then used this rapid zinc exchange capability of our cells to produce viruses from these isotopically enriched zinc cells. The experimental protocol is depicted in Figure 5a, where cells were first transfected with the pNL4.3 plasmid and then the medium was changed twice every 6 h with the isotopically enriched zinc medium. As a result, the produced viral particles would have the same isotopic composition of the producing cells. We employed this strategy because we noted that the presence of ZnCl_2_ in the culture medium drastically affected the efficiency of transfection with calcium phosphate. The viruses were harvested 48 h post transfection and concentrated using a sucrose cushion as described earlier. The purified viruses were subjected to an investigation of (1) the expression level of p24 (Figure 5b), which determines the quantity of produced viruses, (2) the size measured through transmission electron microscopy analysis (Figure 5c,d), and (3) the ability to infect reporter cells carrying the luciferase gene under the control of the Tat promoter (Figure 5e). For all these experiments, viruses were produced using 100 µM of naturally distributed ZnCl_2_ (nat.) as a reference.

First, the expression level of the capsid protein p24, which shares the same precursor polyprotein with NCp7, was generally identical in all conditions (Figure 5b). Therefore, there seemed to be no significant isotopic effect on the overall production of HIV-1 proteins. Secondly, regarding virus morphology, the TEM images showed homogeneous particles in all experimental conditions, with no apparent difference in their average size (Figure 5c,d). As for the most sensitive test in our analysis, the ability to infect TZM-bl cells, a slight effect was noticeable in one of the virus dilutions. Specifically, in the condition where the virus was diluted twice, there was approximately half of the luciferase signal compared with the concentrated virus (compare Figure 5f with Figure 5e). Moreover, it appears that the viruses produced with enriched isotopes (^64^Zn, ^66^Zn, ^67^Zn, and ^68^Zn) generated slightly higher signals than the viruses produced with a natural concentration of the zinc isotopes. However, it remained uncertain whether this phenomenon was due to slightly higher virus production or a minor difference in infectivity. In summary, these experiments suggest that the use of enriched zinc isotopes can selectively label the NCp7 protein in a viral particle without compromising its activity or viral production. This approach holds promise for tracking the fate of this protein in newly infected cells.

## 3. Discussion

The investigation of isotopic composition variations in biology has garnered increasing interest, as isotopic fractionation can arise from biological processes and be a biomarker of physiological changes or pathological conditions [14,15]. In addition, the use of stable isotopes addresses fundamental questions about metabolism, cell signaling, protein dynamics, and other biological processes [17,18,19,20,21,22]. Most importantly, the use of stable isotopes as tracers assumes that the isotopic nature does not influence the chemical reactivity of the element [21,29]. In the present study, we challenged this presumption within the exceptionally delicate framework of viral replication by investigating the correlation between zinc isotopes and the zinc-binding structural protein NCp7 of HIV-1.

The manipulation of zinc by viruses remains poorly elucidated to date, despite the fact that numerous viruses encode proteins that bind zinc for structural or enzymatic functions. A bioinformatics analysis of 226 distinct viral genomes revealed that the majority of viral strains (72%) expressed zinc-binding proteins, indicating the significance of this essential metal ion to viruses [32]. Furthermore, 77% of the zinc-binding domains within viral proteomes belong to the zinc finger type, recognized as the most highly conserved fold. Among these viral proteins, those with a structural role within viral particles are only found in a limited number of viruses. This is the case for the nucleocapsid of retroviruses, such as NCp7 for HIV-1. This is also true for the nucleocapsid of some arenaviruses (Guanarito mammarenavirus, Junin mammarenavirus, Lassa virus, and Lymphocytic choriomeningitis virus) [32]. A few other examples involve capsid proteins in some plant and insect viruses (Caulivirus, Begomovirus, and Baculovirus). In summary, although zinc plays an important role in viral replication, its involvement in the core of the virion particle remains relatively limited to a subset of viruses including retroviruses.

In the context of HIV-1 viral particles, it is noteworthy that additional zinc-binding proteins, namely Vif, Tat, and integrase, are also present within the virions, albeit in significantly lower quantities compared with NCp7 [12]. In our study, we have demonstrated that high viral production results in the accumulation of zinc in HIV-1 particles (Figure 2). Indeed, we substantiated through ICP-MS analysis that the zinc concentration within the HIV viral particle amounted to approximately 16 mM (~40,000 atoms per particle), potentially rendering it one of the most concentrated inorganic elements in the HIV-1 virion. Our experiments on HEK293T cells transfected with the pNL4-3 plasmid showed that the virions captured up to 10% of the amount of cellular zinc within 48 h. More importantly, by using mutants of NCp7, we have provided evidence that this zinc uptake was merely due to the properties of NCp7 and confirmed that the contributions of Vif, Tat, and integrase proteins to the zinc capture within HIV-1 particles remained low. Such data were a prerequisite to establish a potential isotopic fractionation associated with the presence of NCp7 within the viral particle.

As mentioned earlier, a significant amount of cellular zinc is captured by HIV-1 virions. The question of whether this uptake of zinc by the produced viral particles can cause a zinc deficiency in the organism remains to be determined. However, it is worth noting that zinc deficiencies often stem from the inflammatory processes linked to viral infections, as evidenced notably in hospitalized patients with SARS-CoV-2 [33,34]. Often, viral infections not only increase the requirement for micronutrients but also result in their depletion, thereby leading to deficiencies that can be compensated by the supplementation of micronutrients [35].

**Figure 6:**
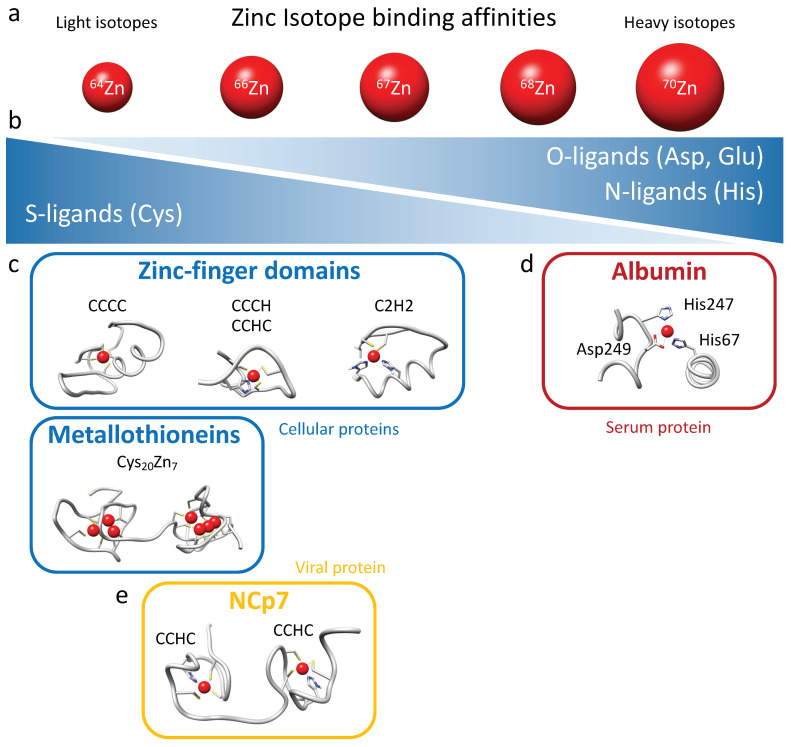
Mechanistic model for the protein-dependent isotopic fractionation of zinc between cellular, medium, and virus compartments. (**a**) The five isotopes of zinc (64, 66, 67, 68, and 70) are depicted from the lightest (left) to the heaviest (right) in red. (**b**) Below, the relative affinities of zinc ligands are illustrated, with S-ligands having higher affinities for the lighter isotopes, while O-ligands and N-ligands prefer the heavier isotopes. (**c**) The major cellular proteins are represented in rounded blue rectangles, including the most common zinc finger domains (PDB: 1GAT, 1UEJ, and 1ZNF) and metallothioneins (PDB: 4MT2). (**d**) The zinc-binding domain of the predominant ligand protein in the serum is depicted in a rounded red rectangle (Albumin, PDB: 1IJF). (**e**) The viral HIV-1 protein NCp7 is represented in an orange rounded rectangle (PDB: 1ESK). The positions of each protein along the horizontal axis illustrate their preference for either light or heavy isotopes.

The isotopic fractionation of zinc isotopes in cells is influenced by coordination chemistry [28]. Heavier isotopes have higher bond energies, and thus they tend to be enriched in the strongest ligand bonds. Therefore, cysteine (S-ligands) preferentially binds lighter zinc isotopes, while histidine (N-ligands) and aspartate (O-ligands), which generally exhibit stronger binding, preferentially interact with heavier zinc isotopes (Figure 6a,b). Isotopic fractionation is increasingly being investigated in biology research due to the advancement of analytical techniques that can now determine the isotopic fractionation of elements present in extremely low concentrations in samples (to the order of nanograms per gram of sample) [21,22,24]. The presence of zinc in numerous chemical compounds makes the analysis of its isotopic fractionation challenging and laborious. Moreover, in general, zinc exhibits relatively low isotopic fractionation since it is redox-inactive. For these reasons, isotopic fractionation of zinc has never been measured in viruses, not even during viral infection. In the present study, we demonstrated for the first time a significant and reproducible fractionation of zinc between different types of cells, whether they were adherent cells (HEK293T) or CD4 lymphocytes in suspension (Jurkat, CEM-T4, and SupT1), and their respective culture media. All cells tested here were indeed enriched with lighter isotopes of zinc. Zinc isotopic fractionation in cellular zinc uptake can be attributed to a wide range of factors due to the significant number of proteins binding this element. Binding ligands for zinc in proteins include histidine, glutamate, aspartate, and cysteine residues (Figure 6b). Histidine-rich loops typically form the primary zinc-binding sites for ZIPs and ZnTs, while cysteine-rich ligands are involved in zinc binding on metallothionein and zinc finger proteins [36]. Metallothioneins bind up to 7 Zn^2+^ coordinated tetrahedrally using a set of 20 cysteines clustered into two distinct domains separated by a linker: an N-terminal β domain that uses 9 cysteines to bind 3 metals, and a C-terminal α domain that binds 4 metals with 11 cysteines (Figure 6c) [37,38]. Cellular zinc finger domains exhibit significant variability in their composition of cysteine and histidine residues, albeit with a majority of cysteines participating in Zn^2+^ coordination (CCCC, CCHC, CCCH, and C2H2 motifs) [6,7,8]. Therefore, considering the abundance of metallothioneins, the composition of zinc finger motifs, and the quasi-absence of intracellular free zinc, it follows that intra-cellular zinc predominantly binds to cysteine residues in proteins and thus favors fractionation toward light isotopes in cells (Figure 6). The variability in zinc finger composition and the predominance of metallothioneins play a critical role in tissue- or cell-specific isotopic fractionation. This phenomenon probably accounts for the enrichment of lighter isotopes in total intracellular zinc compared with the surrounding media, assuming that serum proteins predominantly bind zinc via histidine and aspartate residues.

In the serum of mammals, approximately 75–80% of Zn^2+^ is bound to albumin, accounting for as much as 98% of the exchangeable fraction of Zn^2+^ in blood [39,40,41]. The principal binding site, also called the “multi-metal binding site”, is a tetrahedral coordination of Zn^2+^ by three amino acid residues (His67, His247, and Asp249; Figure 6d), as inferred from X-ray crystallography of human (HSA) and equine serum albumins [40]. Serum albumin effectively acts as an extracellular “zinc buffer” that controls the concentrations of “free” Zn^2+^ ions that are available to other serum proteins or for cellular uptake through membrane-bound zinc transporters [42]. The presence of albumin as the primary zinc binder in serum, through histidine and aspartate residues, provides an explanation for the fractionation toward the heavier zinc isotopes observed in the culture medium (Figure 6d).

Our investigation sought to ascertain whether the absorption of zinc by HIV-1 viral particles exhibits any discernible isotopic fractionation with producing cells. While a distinct fractionation was observed between the cells and growth media, attributable to evident zinc-ligand differentiations, no significant disparity was noted between the HIV-1 particles and producer cells. This outcome was likely influenced by the minimal distinction in binding activity between the CCHC motifs of NCp7 and those of intracellular zinc-fingers proteins harboring CCCC, CCHC, CCCH, and C2H2 motifs and metallothioneins (Figure 6c,e). Nevertheless, a clear distinction was observed between the HIV-1 particles and culture media.

In the absence of treatment, HIV infection progresses through three successive stages: the primary infection (a few weeks), a lengthy latency phase (10–12 years) without symptoms, and then the AIDS phase [9,43,44]. Viral replication is most intense in the initial and final stages of the infection. Investigating the isotopic fractionation of zinc at these three stages would determine whether viremia alone is sufficient to alter the isotopic ratios in the serum through recruiting light isotopes in the HIV-1 viral particles. Additionally, the death of the CD4 cells during the AIDS phase triggers the release of intracellular zinc, and this event could also contribute to changes in the serum isotopic ratios. This would represent a novel instance of fractionation associated with a pathological condition. These hypotheses await further in vivo investigations.

To the best of our knowledge, there have been no prior reports, particularly in the context of zinc and other transition metals, regarding the investigation of isotopic influences on biological or biochemical functions. Here, we investigated a possible isotopic effect of zinc on HIV-1 viral fitness. We examined whether the loading of HIV-1 virus-producing cells with different zinc isotopes resulted in alterations in the quantity, morphology, and infectivity of the produced particles. It can be postulated that changes in atomic size or nuclear spin could have subtle but detectable effects in a highly sensitive system. HIV-1 viral replication involves the protein NCp7 at numerous key stages, and point mutations in zinc fingers can have deleterious effects on the quantity, morphology, and infectivity of the produced particles [10,11,30].

All zinc isotopes have zero nuclear spin (even atomic numbers), except for the zinc isotope 67 (^67^Zn). Isotopes with nonzero nuclear spin possess an intrinsic magnetic moment that creates a local magnetic field around the nucleus, which can influence interactions with other atoms or molecules during the formation of chemical bonds. These magnetic interactions can have subtle yet significant effects on the chemical properties of the bond. Therefore, particular attention must be directed toward the ^67^Zn isotope among all others.

In our experimental protocol, we produced viral particles in HEK293T cells loaded with each of the enriched zinc isotopes. Although the isotopic purity was not absolute, this allowed us to load the cells with 97%, 96%, 86%, 94%, and 66% of isotopes ^64^Zn, ^66^Zn, ^67^Zn, ^68^Zn, and ^70^Zn, respectively, and to compare them to cells loaded with naturally distributed zinc. Although no significant alteration in particle morphology or quantity was observed, subtle variations in HIV-1 infectivity were detected between the enriched isotopes and natural isotopes. In the field of virology, these changes may not be considered drastic, but they could indicate a minimal influence of the isotope composition on NCp7 activity. Further experiments using recombinant purified proteins saturated with various zinc isotopes could provide further insights into the potential impact of isotopes on biological activity. To conclude, our data clearly provides evidence that the use of zinc isotopes as tracers for viral replication or any other research on cellular metabolism appears to be reasonable since these effects are minimal. Approaches in elemental imaging using these zinc tracers could potentially enable the visualization of the fate of zinc initially contained within the viral particle inside infected cells.

## 4. Materials and Methods

### 4.1. Materials

The Jurkat, SupT1, CEM-T4, and HEK293T cells lines used in this study were obtained from ATCC. The TZM-bl cells were obtained from the NIH AIDS Reagent program (Manassas, VA, USA). The cell culture media and supplements, NuPAGE 4–12% bis–Tris polyacrylamide gels, MOPS, and MES SDS running buffer were purchased from Life Technologies (ThermoFisher Scientific, Waltham, MA, USA). Fetal calf serum (FCS), sucrose, NaCl, Tris, DMSO, EDTA, Triton X100, glycerol, and DTT were purchased from Merck (Darmstadt, Germany). The microplate readers (FLUOSTAR OPTIMA and LUMISTAR OPTIMA) were from BMG Labtech (Champigny-sur-Marne, France). The plasmid pNL4.3 and anti-p24 antibodies were obtained from the NIH AIDS Reagent program. The mutant pNL4.3 plasmids were given by D. Muriaux and described in [30]. The different isotopically enriched forms of zinc were purchased in metallic form from Isoflex (^64^Zn, ^66^Zn, and ^68^Zn) and Eurisotop (^67^Zn and ^70^Zn). The metal form of natural zinc was purchased from Merck (#31653).

In order to prevent sample contamination during collection, all plastic and Teflon equipment (Falcon tubes, tips, and ultra-centrifugation tubes) were pre-cleaned in a cleanroom facility with hydrochloric acid for a minimum of 48 h, followed by rinsing and drying. Chemical processing was carried out in a clean room equipped with a reverse filtration system comparable to a biosafety level 3 containment laboratory, specifically designed to protect samples from external contaminants and pollution. All the reagents used were purified by sub-boiling distillation, and appropriate dilutions were made with 18.2 MΩ cm-grade MilliQ water.

### 4.2. Cell Culture

The adherent cells (HEK293T and TZM-bl) were grown and maintained in 75 cm^2^ plates in Dulbecco’s Modified Eagle Medium (D-MEM). The cells in suspension (Jurkat, SupT1, and CEM-T4 cells) were cultured in Roswell Park Memorial Institute medium (RPMI). Media were supplemented with 10% fetal calf serum, 100 μg/mL streptomycin, 100 U/mL penicillin, and 2 mM L-glutamine. Only for the RPMI medium were 1 mM pyruvate and 10 mM HEPES added. The cells were cultivated at 37 °C in a humidified atmosphere containing 5% CO_2_.

### 4.3. HIV-1 Production in HEK293T for ICP-MS Analysis

HIV-1 viral particles were generated through transient calcium phosphate transfection of HEK293T cells with pNL4.3 plasmid DNA (wild type or mutants) in a biosafety level 3 containment laboratory. The HEK293T cells were seeded at a density of 9 × 10^6^ cells per plate 15 cm in diameter the day before transfection. Each experimental condition required 8 plates to produce a sufficient quantity of virus for zinc isotopic fractionation measurement. A single plate was sufficient for a simple measurement of the total zinc quantity. Twenty-three micrograms of plasmid were transfected with calcium phosphate precipitation. The medium was then replaced with fresh medium 6 h later. Two days after transfection, both the medium and cells were collected. The supernatant was collected and centrifuged at 4000× *g* for 10 min to eliminate cellular debris and then ultracentrifuged at 110,000× *g* using a Beckman SW32 rotor for 1 h and 30 min on a TNE sucrose cushion (10 mM Tris, 100 mM NaCl, and 1 mM EDTA) with 20% sucrose to pellet the virus. An aliquot of the supernatant after ultracentrifugation was collected, and the virus pellet was resuspended in ultrapure water. In parallel, the cells were detached by scrapping in 1× PBS and washed by centrifugation at 500× *g* for 10 min. The cellular pellet was resuspended in ultrapure water. The supernatant, virus, and cells were either aliquoted for biochemical analysis or mixed in equal volumes with 13 N nitric acid in a Teflon beaker (Savillex^®^) for ICP-MS measurements. Nitric acid was used to inactivate the samples and remove them from the biosafety level 3 containment laboratory. To quantify the production of HIV-1 in HEK293T cells, we performed an anti-p24 ELISA (HIV-1 Gag p24 DuoSet ELISA, Biotechne) following the manufacturer instructions.

### 4.4. Analyses of Zinc Concentrations through ICP-MS

All the samples coming out of the biosafety level 3 laboratory in concentrated nitric acid media were treated for elemental and isotopic analysis in a metal-free clean-room laboratory under positive air pressure to prevent contamination of the surrounding area. The culture medium, cells, and the viral fraction were digested in HNO_3_ 15 N in Savillex^®^ beakers at 120 °C for 1 day. After evaporation to dryness, they were digested once again in a mixture of HNO_3_ 15 N and H_2_O_2_ (30%) at 120 °C for 1 day to ensure complete decomposition of the organic matter. The mixture could be very reactive, leading to the subsequent creation of nitric and carbon-based fumes that needed to be regularly evacuated to avoid any overpressure, particularly in the early hours of the digestion. After complete dissolution, the samples were dried down and taken up in HNO_3_ 0.5 N media, from which an aliquot corresponding to 2% of the sample was collected for elemental concentration measurements. The remaining samples were reserved for chemical purification of the zinc prior to isotopic composition measurement.

The concentration of zinc was determined with inductively coupled plasma mass spectrometry (ICP-MS) with an Agilent 7500 Series ICP-MS or a Thermo Scientific iCAP Q ICP-MS at LGL-TPE. The concentrations were determined using calibration curves based on Zn elemental solutions. The instrumental drift over the course of the analytical session and matrix effects were monitored and corrected using these standard solutions combined with the measurement of indium as an internal standard.

### 4.5. Analyses of Zinc Isotopic Compositions through MC-ICP-MS

Zinc was isolated and purified by ion exchange chromatography using a Bio-Rad column filled with 0.5 mL of AG 1-X8 (200–400 mesh) anionic resin according to a procedure modified from [45] and described more in detail in [46]. Briefly, the samples were loaded onto the column in HBr 1.5 N media. After elimination of the sample matrix with 2 mL of HBr 1.5 N, zinc was eluted with 5 mL of HNO_3_ 0.5 N. The procedure was repeated twice to ensure the highest purity for better isotope ratio measurement.

The zinc isotopic compositions were measured with multi-collection inductively coupled plasma mass spectrometry (MC-ICP-MS) using Nu Plasma MC-ICP-MS (Nu Instrument, Nu Plasma LR) and Thermo Scientific Neptune Plus MC-ICP-MS. As described in [47], instrumental mass discrimination and temporal drift were corrected with exponential law using the addition of copper as an internal standard, combined with a sample-standard bracketing technique. The isotopic compositions are reported in conventional delta notation (expressed in ‰) relative to the international isotopic standard solutions JMC 3–0749L Johnson Matthey Royston, UK (JMC Lyon):

Δ^x^Zn (‰) = [(^x^Zn/^64^Zn)_sample_/(^x^Zn/^64^Zn)_standard_ − 1] × 1000 (with ×  =  66 or 68).

The ∆^66^Zn values represent the differences between two different δ^66^Zn as follows:

∆^66^Zn_cells_ = δ^66^Zn_cells_ − δ^66^Zn_medium_

∆^66^Zn_virus_ = δ^66^Zn_virus_ − δ^66^Zn_medium_

All zinc isotopic data in the current study follow the theorized trend for mass-dependent isotopic fractionation, and therefore only δ^66^Zn is discussed. Wherever possible, the zinc isotopic compositions were analyzed multiple times (three replicate analyses for most samples), and the error values for all isotopic analyses have been reported as two times the standard deviation (2σ), in line with the convention.

### 4.6. HIV-1 Production in HEK293T in the Presence of Enriched Zinc

For each enriched isotope spike and natural zinc, metal zinc was transformed in ZnCl_2_ with ultrapure hydrochroric acid. The concentration and isotopic composition of the zinc was measured with single-quadripole ICPMS (iCAP-Q), and the volumes were adjusted to reach a 100 mM concentration. The isotopic composition is given in Table S1. The day prior to transfection, the HEK293T cells were seeded at 6.5 × 10^6^ cells per plate 10 cm in diameter. The following day, the culture medium was changed, and the cells were transfected 2 h later with 10 μg of pNL4.3 plasmid using calcium phosphate precipitation. After 6 h, the medium was replaced with a medium containing either 100 μM of isotopically enriched or naturally abundant zinc. The medium was changed once more after 6 h with the same media, and the cells were incubated for 48 h at 37 °C with 5% CO_2_.

### 4.7. Quantification of Infectious HIV-1 Produced by HEK293T Cells

The viral particles produced by HEK293T cells were analyzed for their capacity to infect TZM-bl cells, a cell line that carries the luciferase gene under the control of the HIV-1 promoter (LTR). The TZM-bl cells were seeded in 96 well plates. The following day, the virus was serially diluted (1/3) in triplicate and added to the cells, which were then incubated for 48 h at 37 °C with 5% CO_2_. The cell density in each well was determined using Cell-Titer Fluor (Promega). Then, the cells were lysed, and the luciferase activity was determined using the Luciferase assay system (Promega) following the manufacturer’s protocol. The luminescence was measured using a Tecan luminometer. The luciferase activity was normalized to the cell density for each well.

### 4.8. Transmission Electron Microscopy (TEM) Analysis

The viruses were concentrated from the supernatant on a TNE sucrose cushion as described above and purified over an optiprep gradient (5–20%). The fractions containing the viral particles were concentrated on a TNE sucrose cushion, and the pellet was then resuspended in 20 µL of a fixation solution containing 4% PFA. The suspensions were adsorbed on 200 mesh nickel grids coated with formvar-C for 10 min at RT. Then, grids with the suspension were colored with phosphotungstique acid 2% (Electron Microscopy Sciences, Hatfield, PA, USA) for 2 min and observed on a transmission electron microscope (Jeol 1400 JEM, Tokyo, Japan) equipped with a Gatan camera (Orius 1000) and Digital Micro-graph software (version 1.7).

### 4.9. Protein Extraction and Western Blot

Cellular protein extracts aliquoted for biochemical analysis were treated with lysis buffer (25 mM Tris-HCl, pH 7.8, 2 mM DTT, 2 mM EDTA, 1% Triton X-100, and 10% glycerol). Then, the protein concentrations were measured using a DC protein assay kit (Biorad, South Granville, Australia) in microplate assays. Equal protein amounts (30 µg) were separated in 4–12% Bis-Tris NuPAGE Novex Midi Gels and transferred onto nitrocellulose membranes using an iBlot Dry blotting system (ThermoFisher Scientific). The membranes were probed with anti-p24 primary antibodies and HRP-conjugated anti-mouse secondary antibodies. The chemiluminescence signal was detected using an ECL Select detection kit (GEHealthcare, Chicago, IL, USA) in a Chemidoc Imager (Biorad).

## 5. Conclusions

Zinc plays a pivotal role in HIV-1 replication, particularly through its presence in the zinc finger motifs of the nucleocapsid protein NCp7, a key structural component of viral particles. Using inductively coupled plasma mass spectrometry, we demonstrated a significant capture of cellular zinc by HIV-1 particles and shown that both cultured cells and HIV-1 viral particles preferentially concentrate lighter zinc isotopes over the culture medium. With zinc-enriched isotopes, we observed a rapid exchange of cellular zinc, enabling the labeling of HIV-1 viral particles. As no isotopic effect was detected, these viral particles can potentially be employed in the future for tracking or imaging the fate of zinc within infected cells.

## Data Availability

Requests for further information about resources, reagents, and data availability should be directed to the corresponding author.

## Supplementary Materials

The following supporting information can be downloaded at https://www.mdpi.com/article/10.3390/ijms242015274/s1.
