# Supplementary material for: Zinc Uptake by HIV-1 Viral Particles: An Isotopic Study
Source: Int J Mol Sci. 2023 Oct 17;24(20):15274. doi: 10.3390/ijms242015274 (PMC10607083; doi:10.3390/ijms242015274)
Supplement: Supplementary file 1 [file ijms-24-15274-s001.zip › ijms-2652675-supplementary.pdf]

Supplementary Figure S1

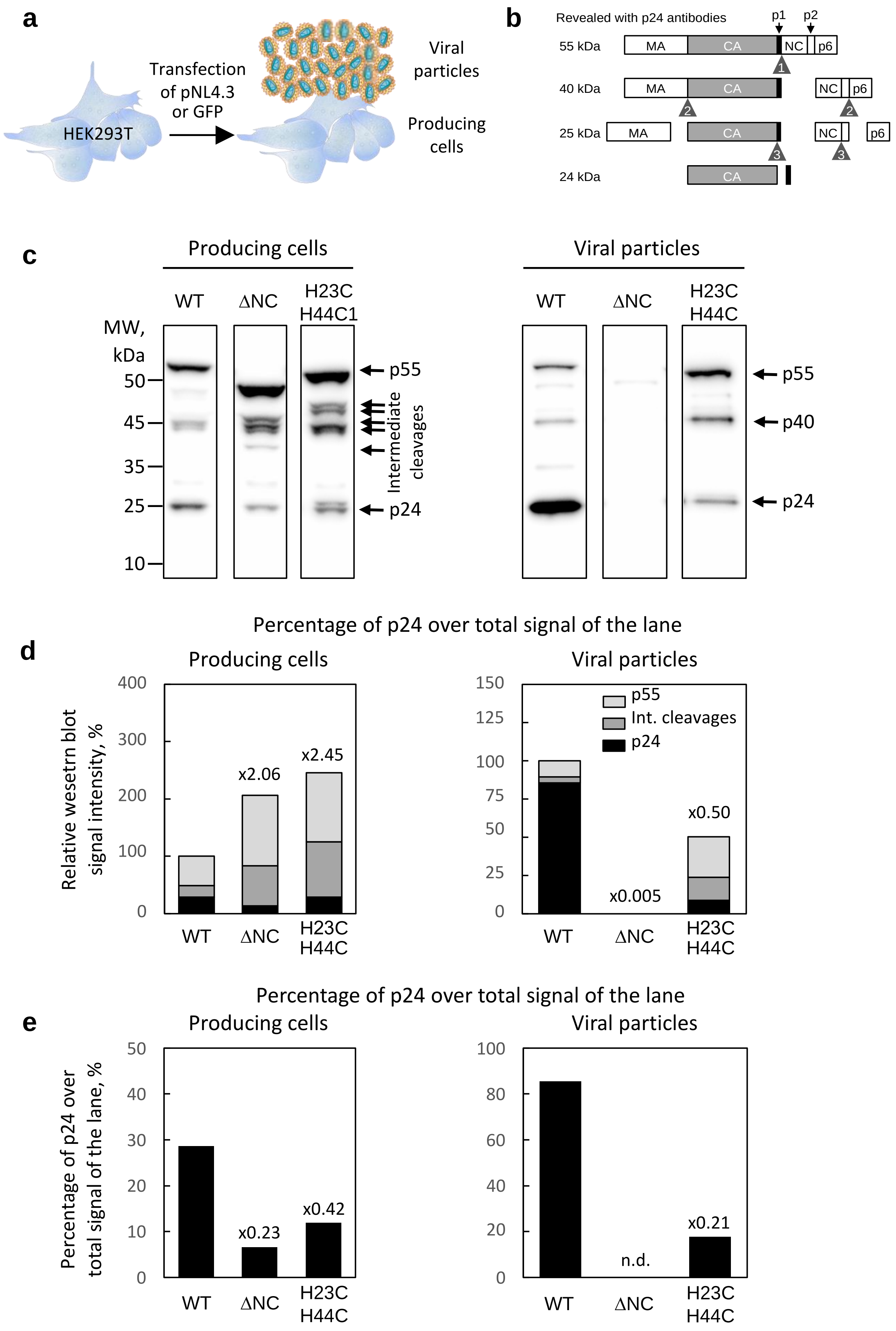

Supplementary Figure S1. Expression levels of GAG proteins after transfection of various pNL4.3 plasmids. (a) Experimental design: Different pNL4.3 plasmids were used in transfection to produce viral particles containing either the wild-type (WT) or a mutated version of NCp7. (b) Maturation scheme of the GAG polyprotein, with the portion revealed by the anti-p24 antibody (CA, p24) shown in gray. The NCp7 mutants include: ΔZF2, deletion of the zinc finger domain 2 (ZF2); ΔNC, deletion of the entire NCp7; H23C, mutation of histidine 23 (ZF1 domain) to cysteine; H44C, mutation of histidine 44 (ZF2 domain) to cysteine; H23C/H44C, double mutation in the ZF1 and ZF2 domains. (c) Intracellular extracts and viral particles were analyzed by Western blot using anti-p24 antibodies, where the major proteins p55, p40, and p24 are indicated by ar-rows. With the different deletions, the sizes of precursor GAG proteins (p55) and partially cleaved forms differ from those of the wild-type track (other arrows). (d) Signal intensities were quantified using Imagelab software and represented in histogram form, with the first track (WT) taken as the reference at 100%. (e) Intensities of p24 (fully matured form of GAG in CA) are de-picted in an independent histogram. The ratio compared to the first track is indicated in the two graphs above the bar.

Supplementary Table S1. Isotopic composition of $ZnCl_2$ solutions derived from metallic zinc

	Isotopic composition				
Type of $ZnCl_2$	^{64}Zn	^{66}Zn	^{67}Zn	^{68}Zn	^{70}Zn
Natural	48,63	27,90	4,10	18,75	0,62
Enriched in ^{64}Zn	99,1	0,69	0,04	0,17	0,01
Enriched in ^{66}Zn	0,70	98,00	1,20	0,09	0,01
Enriched in ^{67}Zn	1,56	3,88	89,60	4,91	0,01
Enriched in ^{68}Zn	0,50	0,70	0,50	98,30	0,001
Enriched in ^{70}Zn	8,36	7,28	1,71	12,45	70,20
